# Expression of the RAE-1 Family of Stimulatory NK-Cell Ligands Requires Activation of the PI3K Pathway during Viral Infection and Transformation

**DOI:** 10.1371/journal.ppat.1002265

**Published:** 2011-09-22

**Authors:** Maria Tokuyama, Clarisse Lorin, Frederic Delebecque, Heiyoun Jung, David H. Raulet, Laurent Coscoy

**Affiliations:** Division of Immunology and Pathogenesis, Department of Molecular & Cell Biology, University of California, Berkeley, Berkeley, California, United States of America; Oregon Health Sciences University, United States of America

## Abstract

Natural killer (NK) cells are lymphocytes that play a major role in the elimination of virally-infected cells and tumor cells. NK cells recognize and target abnormal cells through activation of stimulatory receptors such as NKG2D. NKG2D ligands are self-proteins, which are absent or expressed at low levels on healthy cells but are induced upon cellular stress, transformation, or viral infection. The exact molecular mechanisms driving expression of these ligands remain poorly understood. Here we show that murine cytomegalovirus (MCMV) infection activates the phosphatidylinositol-3-kinase (PI3K) pathway and that this activation is required for the induction of the RAE-1 family of mouse NKG2D ligands. Among the multiple PI3K catalytic subunits, inhibition of the p110α catalytic subunit blocks this induction. Similarly, inhibition of p110α PI3K reduces cell surface expression of RAE-1 on transformed cells. Many viruses manipulate the PI3K pathway, and tumors frequently mutate the p110α oncogene. Thus, our findings suggest that dysregulation of the PI3K pathway is an important signal to induce expression of RAE-1, and this may represent a commonality among various types of cellular stresses that result in the induction of NKG2D ligands.

## Author Summary

Human and mouse cytomegaloviruses (HCMV and MCMV) are members of the Herpesvirus family. Both viruses cause disease in individuals with a compromised immune system, such as transplant patients and AIDS patients. Natural killer (NK) cells are essential players in the immune response against these viruses. NK cells recognize self-proteins, such as NKG2D ligands, that are poorly expressed on healthy cells but are upregulated on cells that are undergoing stress, such as infection and tumor development. The biological processes associated with NKG2D ligand expression in infected cells are unknown. The PI3K pathway, which controls many cellular processes, is activated by a variety of viruses to prime cells for efficient viral replication. We observed that MCMV activates the PI3K pathway and that this activation is required for NKG2D ligand expression. We also found that the expression of NKG2D ligands on cancer cell lines is dependent on this pathway. Our data suggest that NKG2D ligand expression, and thus recognition of infected and cancer cells by NK cells, is associated with a dysregulation in the PI3K pathway.

## Introduction

Natural killer (NK) cells are specialized lymphocytes of the innate immune system that target both tumor cells and virally-infected cells. NK-cell cytotoxicity is regulated by a balance of signaling through inhibitory and stimulatory receptors [1], [2]. Most of the inhibitory receptors generally recognize major histocompatibility complex I (MHC-I) molecules, a set of proteins often downregulated during viral infection or tumorigenesis. Stimulatory receptors recognize a wide variety of self-proteins that are induced upon viral infection or cellular transformation. Together, a net positive signal activates NK cells to secrete proinflammatory molecules TNF-α and IFN-γ, as well as effectors of lysis, granzymes and perforin [3].

NKG2D is a well-studied and potent NK-stimulatory receptor that is expressed on the surface of NK cells, activated CD8 T cells, and subsets of γδ T cells and NKT cells [4]. NKG2D can also function as a co-stimulatory receptor to enhance T-cell activation [5], [6]. The human genome encodes at least seven NKG2D ligands (MICA, MICB, ULBP1-4, and RAET1G), and the mouse genome encodes at least nine NKG2D ligands (MULT-1, H60a-c, and RAE-1α-ε). Although the ligands bind NKG2D with varying affinities, they all trigger NK cell killing of target cells similarly. NKG2D ligand transcripts can be detected in certain cell types or during specific phases of development, but in general, ligand expression is low or absent in healthy cells [4]. However, ligands are induced during various stress conditions including transformation, DNA damage, and viral infection. Accordingly, NKG2D ligands are constitutively expressed on many tumor cell lines and on a large array of tumors including melanomas, leukemias, various carcinomas, and neuroblastomas [7], [8]. NKG2D ligands are also upregulated in cells infected with viruses such as cytomegalovirus (CMV), measles, Influenza A, and respiratory syncytial virus [9], [10]. To counteract this NK recognition, tumors and viruses have evolved ways to shed or block surface expression of NKG2D ligands [11], [12]. In particular, studies using mouse CMV (MCMV) with deletion mutations in genes encoding proteins that block ligand expression have shown that the ability of the virus to evade NKG2D recognition has a significant advantage on viral fitness *in vivo* [13]–[15]. Furthermore, aberrant expression of NKG2D ligands can lead to unwanted NKG2D signaling, which has been implicated in autoimmune diseases, such as rheumatoid arthritis and type 1 diabetes [16]. Therefore, regulation of ligand expression under different conditions is critical to prevent targeting of healthy cells.

Several modes of regulation have been shown for NKG2D ligand expression. At the transcriptional level, expression of human NKG2D ligands MICA and MICB seems to be controlled by heat shock elements in their promoters [17]. Damage of genomic DNA also leads to increased expression of RAE-1, MULT-1, ULBP1-3 and MICA, and RAE-1 induction occurs through the action of ataxia telangiectasia mutated (ATM) and/or ataxia telangiectasia and Rad3-related (ATR), as well as checkpoint effector kinase1 (Chk1) [18]. Additionally, it was reported that c-Myc regulates RAE-1ε at the transcriptional level [19]. At the post-transcriptional level, the expression of MICA and MICB can be inhibited by cellular microRNAs, and MICB expression can also be inhibited by viral microRNAs [20], [21]. Finally, the expression of MULT-1 is regulated post-translationally through ubiquitination [22].

The effect of NKG2D ligand expression on NK cell activity, both *in vitro* and *in vivo*, has been best characterized with the RAE-1 family of mouse NKG2D ligands. Cells that normally do not express NKG2D ligands become highly susceptible to NK cell-mediated lysis *in vitro* when transduced with RAE-1 [8], [23]. Ectopic expression of RAE-1 in tumor cells also results in efficient clearance of tumor cells after subcutaneous transfer *in vivo*. Clearance *in vivo* is mediated by NK cells and in some cases CD8 T cells, despite expression of inhibitory MHC-I molecules in some tumor cells [6], [24]. Together these data demonstrate that RAE-1 expression results in NK-cell susceptibility both *in vitro* and *in vivo*, and highlight the importance of understanding the molecular mechanism of RAE-1 expression.

Despite some evidence showing the role of certain pathways and effector molecules in the expression of NKG2D ligands, much remains to be learned about the process, and uncovering the molecular mechanism that drive expression of each of the NKG2D ligands remains an active area of research in the field. In particular, very little is known concerning the mechanisms of RAE-1 induction in virus-infected cells. CMV infection results in the induction of transcripts encoding numerous NKG2D ligands, including RAE-1, MULT-1, and H60a in the mouse. However, both human and mouse CMV encode proteins that specifically inhibit expression of each of the NKG2D ligands at the protein level, suggesting that NK cell recognition of CMV-infected cells has put evolutionary pressure on the virus to evade this arm of the immune system. The inducibility of RAE-1 in MCMV-infected cells prompted us to use this well characterized virus to investigate the molecular mechanism of RAE-1 induction. Strikingly, our studies showed that virus-induced activation of phosphatidylinositol-3-kinase (PI3K) is essential for the induction of the RAE-1 family of mouse NKG2D ligands. Further studies demonstrated that PI3K is also important for the maintenance of RAE-1 and MULT-1 expression on transformed cells, showing the breadth of our findings. These results suggest that activation of the PI3K pathway, which occurs in cells infected with numerous viruses and in cancer cells, represents a common signal for regulating RAE-1 expression. Finally, the effect of PI3K inhibition on MULT-1 expression also reveals the possibility that PI3K activation may play a role in regulating expression of other NKG2D ligands in cells infected with other viruses and other pathologic states such as inflammatory diseases.

## Results

### RAE-1 mRNA and protein are induced upon MCMV infection

**Figure 1:**
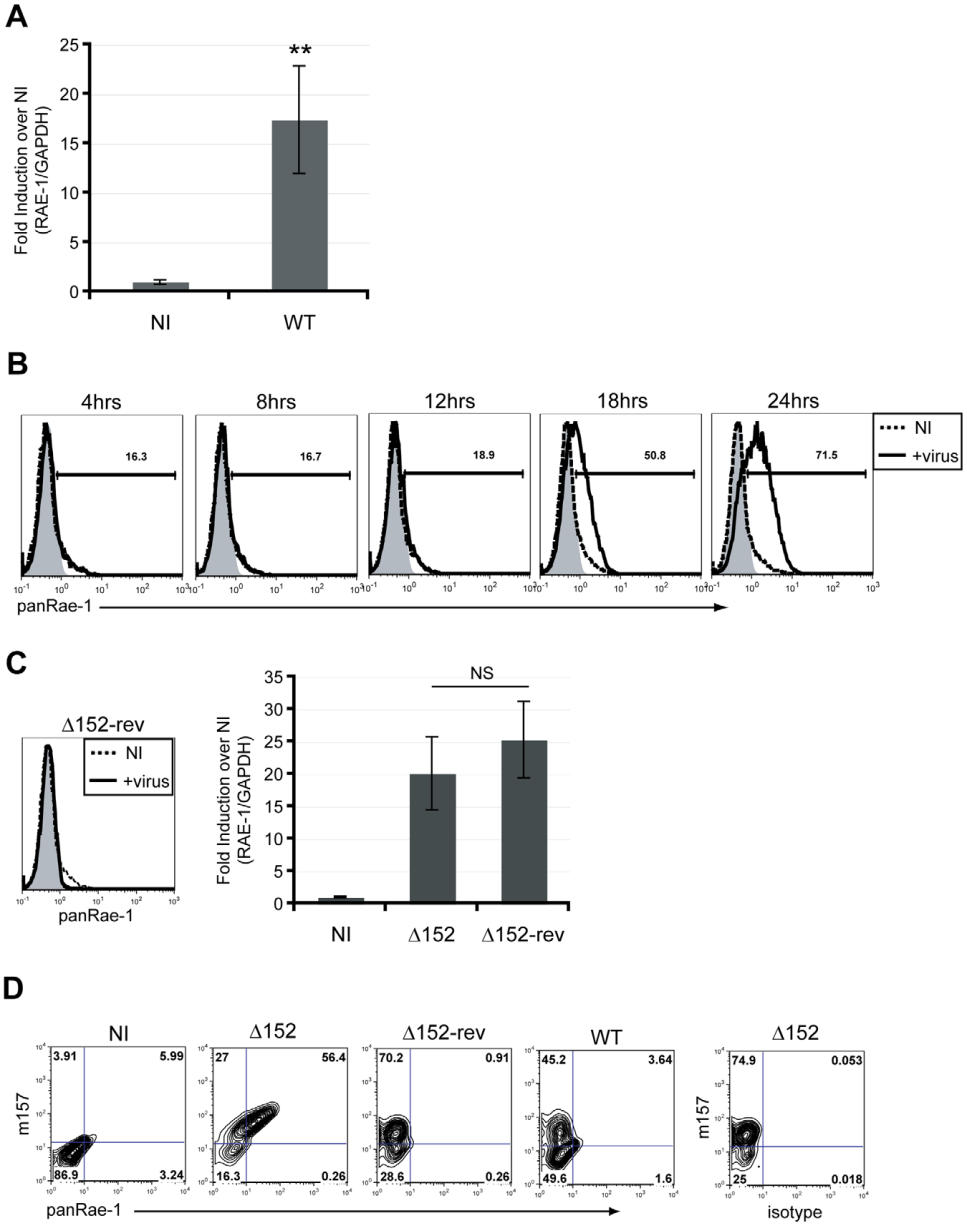
RAE-1 mRNA and protein are induced upon infection of mouse fibroblasts. **A**) The level of RAE-1 mRNA induction was determined in MCMV-infected fibroblasts (WT) at 24 hrs post-infection (pi) using RT-qPCR. Fold induction was determined by normalizing the infected samples to uninfected samples (NI). SD and statistical significance were determined from five independent samples. **p<0.01 compared to NI. **B**) RAE-1 expression on the surface of fibroblasts infected with MCMVΔ152 for 4, 8, 12, 18 and 24 hrs pi was determined using a pan RAE-1 antibody. The data shown are gated on live, 7AAD negative cells. Histograms show isotype control (shaded gray), uninfected (dashed black), and infected cells (solid black). **C**) RAE-1 surface expression on fibroblasts infected with MCMVΔ152 revertant (Δ152-rev) was analyzed using a pan RAE-1 antibody at 24 hrs pi. The level of RAE-1 mRNA in cells infected with Δ152 and Δ152-rev was quantified by RT-qPCR. Fold induction was determined by normalizing the infected samples to uninfected samples (NI). The data in the figure are representatives of three independent experiments. NS, not statistically significant. **D**) RAE-1 expression was determined in infected versus uninfected cells. Cells were infected with Δ152, Δ152-rev or WT virus and co-stained for MCMV m157 and RAE-1 at 24 hrs pi.

Most cell lines constitutively express varying levels of RAE-1 at the cell surface [8]. Because most cells *in vivo* generally express very low levels of NKG2D ligands, if any at all, we utilized established mouse-tail fibroblasts that do not express RAE-1 at the surface to investigate the mechanism of RAE-1 induction upon MCMV infection. These fibroblasts have previously been used to demonstrate RAE-1 induction upon activation of the DNA damage response [18]. Upon infection of fibroblasts with MCMV for 24 hours, there was a significant induction of RAE-1 expression at the RNA level (Fig. 1A). In order to further observe RAE-1 induction at the cell surface, we utilized a MCMV mutant (MCMVΔ152) lacking the m152 gene, which encodes an immune evasin that downregulates RAE-1 protein. Using this virus, we observed RAE-1 surface expression starting 18 hours post-infection with an even higher expression at 24 hours post-infection (Fig. 1B). Importantly, RAE-1 surface expression was not observed upon infection with the revertant virus (MCMVΔ152-rev) at 24 hours post-infection, despite no significant difference in the levels of RAE-1 mRNA induction (Fig. 1C).

Although previous studies have demonstrated the ability of MCMV to induce RAE-1 expression, it was not determined whether induction occurs specifically in infected cells or also in neighboring uninfected cells by an indirect mechanism. To address this question, we distinguished infected versus uninfected cells by staining cells with an antibody specific for an MCMV protein, m157, that is expressed at the cell surface of infected cells [25]. Co-staining experiments demonstrated that RAE-1 induction occurs only in infected cells, suggesting that RAE-1 induction is a direct consequence of infection (Fig. 1D). The m157-positive cells that express RAE-1 at low levels are most likely cells that were recently infected in the cultures and have not had sufficient time to upregulate RAE-1. In subsequent experiments, we determined which events associated with the viral life cycle are necessary for RAE-1 induction.

### Viral gene expression is necessary for RAE-1 induction

Upon entry, MCMV initiates a sequence of well-characterized events including transcription of immediate early and early genes, which are essential for viral replication and for the activation of cellular pathways aimed at priming the cell for efficient viral replication [26]. Expression of these early genes is also required for the expression of late genes and subsequent packaging and budding of the virus [27].

**Figure 2:**
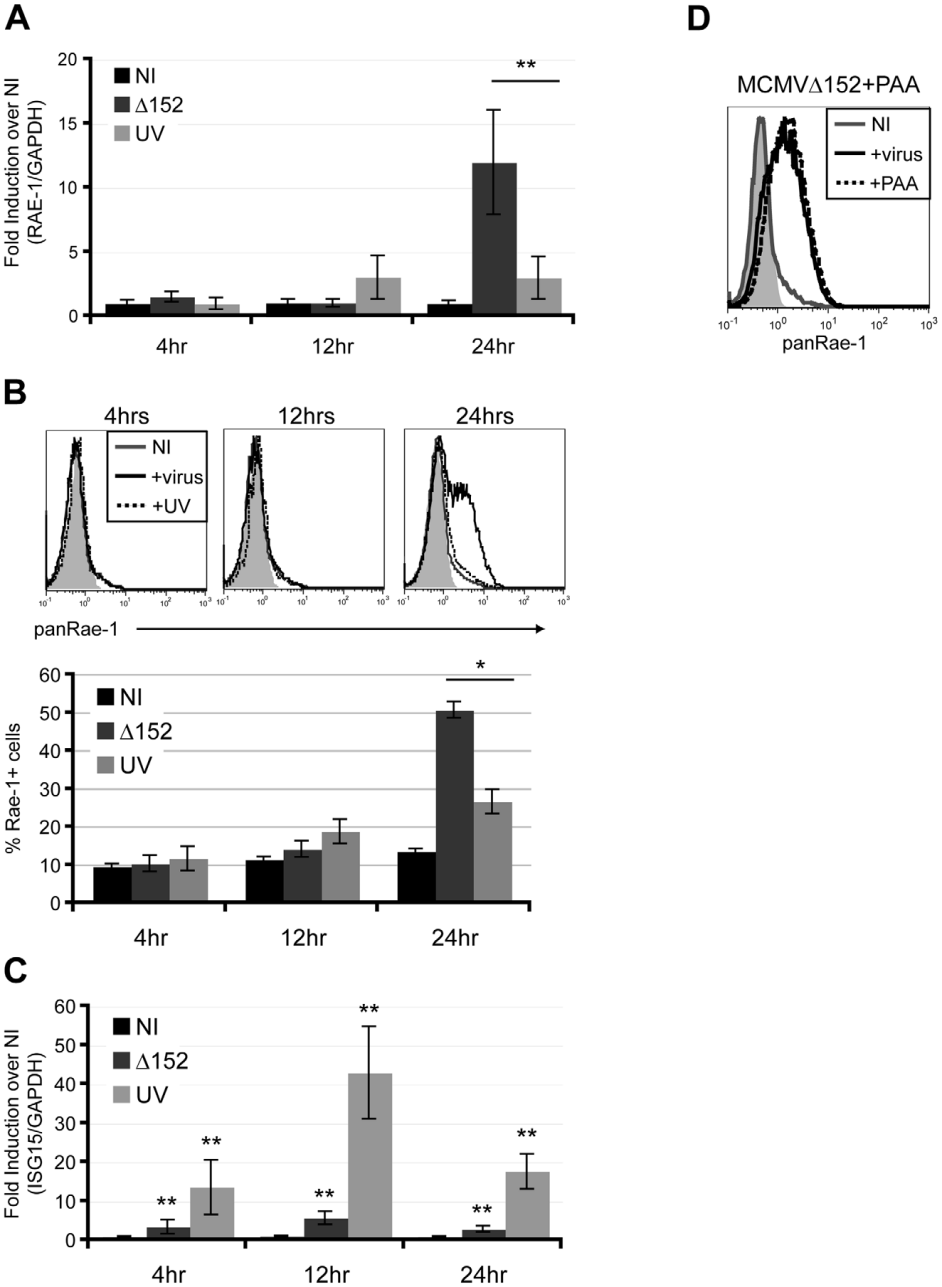
Viral early gene expression is necessary for the induction of RAE-1. Fibroblasts were infected with either MCMVΔ152 (Δ152) or UV-inactivated MCMVΔ152 (UV) for 4, 12, and 24 hrs, and RAE-1 mRNA expression (**A**), RAE-1 cell surface expression (**B**), and ISG15 mRNA expression (**C**) were determined using RT-qPCR and flow cytometry. Fold induction was determined by normalizing the infected samples to uninfected samples (NI). SD and statistical significance were determined from three independent samples. *p<0.05 and **p<0.01. **D**) Fibroblasts were infected with MCMVΔ152 in the presence of 100 ug/ml PAA and stained with a pan RAE-1 antibody. Histograms show isotype control (shaded gray), uninfected (solid gray), MCMVΔ152-infected (solid black), and cells infected with MCMVΔ152 in the presence of PAA (dashed black). The histogram is a representative figure of three independent experiments.

To investigate whether expression of viral genes is necessary for RAE-1 induction, fibroblasts were infected with either MCMVΔ152 or UV-inactivated MCMVΔ152 for 24 hours. UV inactivation significantly impaired the ability of MCMVΔ152 to induce expression of RAE-1 both at the RNA and protein levels throughout the course of the infection (Fig. 2A and B). Interferon-Stimulated Gene 15 (ISG15) expression was significantly induced upon infection by both MCMVΔ152 (Δ152) and UV-inactivated virus (UV) (Fig. 2C), indicating that neither viral entry nor activation of the interferon response was affected by the UV treatment. As a control, MCMV early gene 1 (e1) product was PCR amplified using viral genomic DNA extracted from either MCMVΔ152 or UV-inactivated MCMVΔ152, and no amplification was observed from the UV-inactivated viral genomic DNA (data not shown). UV-inactivation was also confirmed by the lack of plaque forming units in the supernatant of cells infected with UV-inactivated virus for 24 hours (data not shown).

We next determined whether viral DNA replication is required for the induction of RAE-1 using phosphonoacetic acid (PAA), a chemical inhibitor that binds to the viral DNA polymerase and blocks CMV viral replication [28]. Infection of fibroblasts with MCMVΔ152 for 24 hours in the presence of PAA did not inhibit RAE-1 induction, indicating that viral DNA replication and late gene expression are dispensable for RAE-1 induction (Fig. 2D). Altogether, our results suggest that expression of viral genes at an early stage upon infection prior to viral replication is necessary for the induction of RAE-1.

### The DNA damage response is not required for RAE-1 induction by MCMV

Stress-induced activation of the DNA damage response, through the action of ATM or ATR and Chk1, has been implicated in the induction of RAE-1 and other NKG2D ligands [18], [29], [30]. Additionally, CMV has been shown to manipulate the DNA damage response [31], [32]. Therefore, we tested the role of the DNA damage response in the induction of RAE-1 in MCMV-infected cells by infecting fibroblasts for 24 hours in the presence or absence of specific inhibitors of the DNA damage response pathway. Inhibition of Chk1 using SB218078 and UCN-01, or inhibition of ATM/ATR using caffeine did not affect RAE-1 induction upon MCMV infection, indicating that activation of the DNA damage response is not required for MCMV-induced RAE-1 expression (Fig. S1).

### RAE-1 induction requires activation of the PI3K pathway

**Figure 3:**
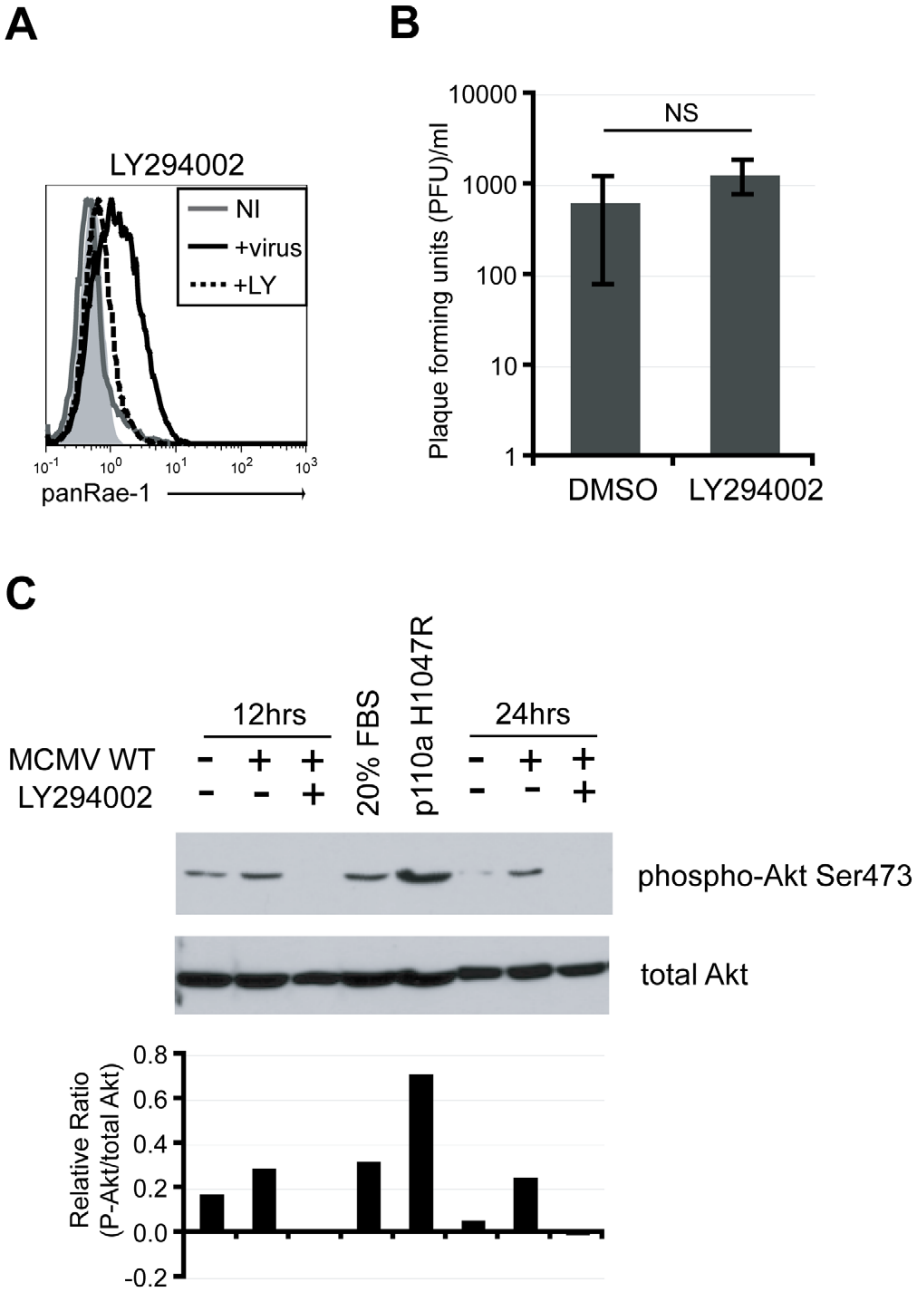
PI3K pathway is required for RAE-1 induction in MCMV-infected cells through the activation of Akt. **A**) Fibroblasts infected with MCMVΔ152 in the presence of 20 uM LY294002 were stained for RAE-1 at 24 hrs pi. Histograms show isotype control (shaded gray), uninfected (solid gray), MCMVΔ152-infected (solid black), and MCMVΔ152-infected cells in the presence of LY294002 (dashed black). The histogram is a representative figure from three independent experiments. **B**) Plaque forming units (PFU)/ml were determined in supernatants of infected cells in the presence of DMSO or LY294002. SD and statistical significance were determined from three independent experiments. NS, not statistically significant. **C**) Whole cell lysates from fibroblasts infected with MCMV WT in the presence of DMSO or LY294002 were obtained at 12 and 24 hrs pi. Lysates were also obtained from cells treated with 20% FBS or cells overexpressing p110α H1047R. All samples were blotted for phospho-Akt S473 and total Akt. The relative ratio of P-Akt to total Akt from the blot was determined. The data is a representative experiment from ten independent experiments.

Many cellular pathways are activated early on upon viral infection to achieve a state of pro-survival and increased cellular proliferation for optimal replication and production of progeny virus [33]. Common cellular pathways activated upon viral infections include the mitogen-activated protein kinase (MAPK) and the phosphatidylinositol-3-kinase (PI3K) pathways [34], [35]. The PI3K pathway, in particular, is crucial in controlling cell growth and survival and is a key pathway in promoting cellular transformation, another condition known to trigger NKG2D ligand expression [36]. Because our data suggested that early events upon viral infection are necessary to induce RAE-1, we hypothesized that manipulation of some of these cellular pathways are involved in the induction. To test this hypothesis, fibroblasts were infected with MCMVΔ152 for 24 hours in the presence of known inhibitors of these pathways, and RAE-1 surface expression was analyzed. MCMV-induced RAE-1 induction was not affected by the presence of MAPK inhibitors (Fig. S2). Remarkably however, surface expression of RAE-1 was completely abrogated in the presence of LY294002, a global inhibitor of PI3K that binds to the catalytic domain of the kinase [37] (Fig. 3A). Viral titers in the supernatant collected from cells infected for 24 hours in the presence or absence of LY294002 were not significantly different, indicating that the absence of RAE-1 surface expression was not due to a lack of viral entry and replication (Fig. 3B). Of note, input virus was removed two hours post-infection, and therefore, virus present in the supernatant at 24 hours post-infection is a measure of progeny virus produced as a result of infection and replication. Furthermore, inhibitors were added at two hours post-infection to fresh culture media to prevent possible blockage of viral attachment or entry.

The requirement for active PI3K to induce RAE-1 suggests that MCMV infection activates the PI3K pathway. To determine whether the PI3K pathway is activated upon MCMV infection in our system, cellular lysates from MCMV-infected fibroblasts were analyzed by western blotting with an antibody specific for Akt phosphorylated at Serine 473. As positive controls, whole cell lysates were obtained from cells treated with 20% fetal bovine serum (FBS) or cells stably expressing the catalytic subunit of PI3K (p110α) with an H1047R mutation that renders PI3K constitutively active [38]. Very little Akt phosphorylation was observed in uninfected cells. By contrast, Akt phosphorylation was readily detectable in MCMV-infected cells as well as in cells treated with FBS and cells stably expressing p110α H1047R (Fig. 3C). When cells were infected in the presence of LY294002, the phosphorylated form of Akt was no longer detectable. Altogether, our data indicate that MCMV infection activates the PI3K pathway, in accordance with data obtained with HCMV [39], and that this activation is required for the induction of RAE-1.

### Signaling through p110α PI3K is essential for RAE-1 induction in MCMV-infected cells

There are three classes of enzymes in the PI3K superfamily, class I, II, and III. Akt activation occurs mainly through class I PI3K, and this is critical in regulating cell survival, metabolism, apoptosis, and cell cycle. Class I PI3Ks are heterodimeric molecules composed of a catalytic and a regulatory subunit and are classified into class IA or class IB PI3K. The catalytic subunits of class IA PI3K are p110α, β or δ, whereas class IB PI3K contains p110γ [40]. It is becoming increasingly appreciated that PI3K catalytic subunits play non-redundant roles in regulating the biology of the cell [41]. Thus, we hypothesized that RAE-1 induction upon MCMV infection occurs through a particular PI3K isoform such that a specific signal is required for its expression.

**Figure 4:**
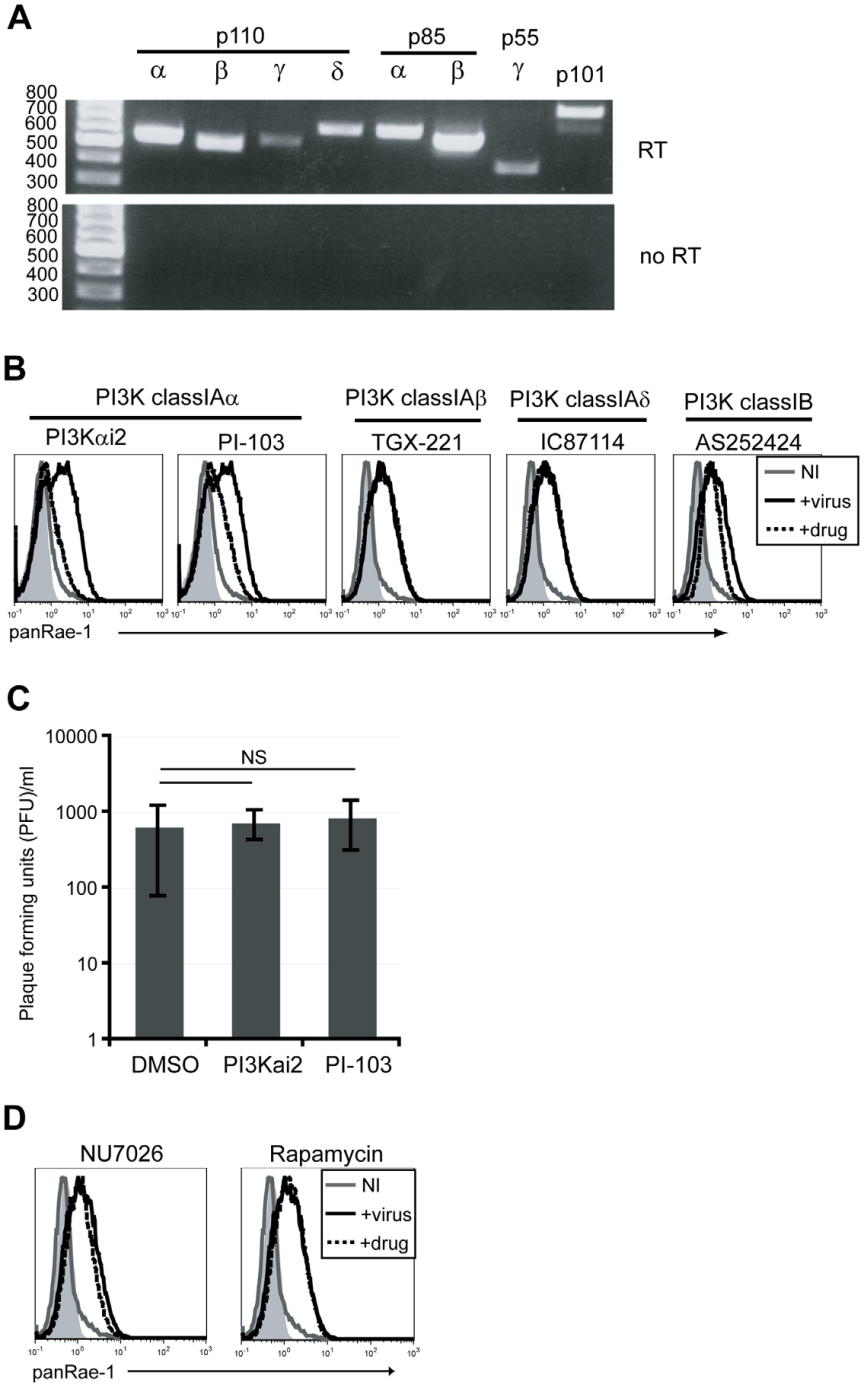
p110α PI3K is specifically involved in the induction of RAE-1 in fibroblasts infected with MCMV. **A**) Expression of PI3K catalytic and regulatory domains of class IA and IB PI3K in mouse fibroblasts at the steady state level was determined by RT-PCR. Top gel shows amplification of fibroblast cDNA and the bottom gel shows amplification of no RT controls. Size of the ladder is indicated in base pairs. **B**) Fibroblasts infected with MCMVΔ152 in the presence 25 uM PI3Kαi2, 1.25 uM PI-103, 5 uM TGX-221, 10 uM IC87114 or 10 uM AS252424 were surface stained for RAE-1 at 24 hrs pi. **C**) PFU/ml was determined in supernatants from cells infected in the presence of DMSO, PI3Kαi2 or PI-103. SD and statistical significance were calculated from three independent experiments. NS, not statistically significant. **D**) Fibroblasts infected with MCMVΔ152 in the presence of 10 uM NU7026 or 100 nM rapamycin were stained for RAE-1 at 24 hrs pi. All histograms show isotype control (shaded gray), uninfected (solid gray), MCMVΔ152-infected cells in the presence of DMSO (solid black) or inhibitors (dashed black). All histograms are representative figures from three independent experiments.

In order to determine which of the PI3K isoforms are involved in RAE-1 induction, we first determined the expression patterns of each of the PI3K catalytic and regulatory subunits in our fibroblasts at the steady state level by RT-PCR analysis. Our analysis showed that all of the catalytic and regulatory subunits were detected to varying degrees in these cells (Fig. 4A). We then employed isoform-specific inhibitors to test the role of each of the class I PI3Ks on MCMV-induced RAE-1 expression. RAE-1 surface expression was greatly diminished when cells were infected in the presence of inhibitors for p110α (PI3Kαi2 and PI-103), but not in the presence of inhibitors for p110β (TGX-221), p110δ (IC87114) or p110γ (AS252424) (Fig. 4B). Similar to treatment with LY294002, treatment with either PI3Kαi2 or PI-103 did not result in a significant change in the viral titer in the supernatant, indicating that the loss of RAE-1 expression in these cells was not due to the lack of viral entry or replication (Fig. 4C).

Compared to LY294002, PI-103 is much more selective for p110α, but at higher concentrations it is still able to inhibit other targets in the pathway, namely DNA-PK and mTORC1 [42]. Therefore, to rule out possible contributions from these molecules on RAE-1 induction, selective inhibitors of DNA-PK and mTORC1 (NU7026 and rapamycin, respectively) were tested in the same assay. Neither NU7026 nor rapamycin treatment inhibited RAE-1 induction, suggesting that indeed RAE-1 induction upon MCMV infection involves signaling specifically through the p110α-containing PI3K (Fig. 4D). These results were further confirmed using a wide range of inhibitor concentrations in MCMV-infected cells (Fig. S3).

### p110α PI3K is also important for the maintenance of RAE-1 and MULT-1 on transformed cells

**Figure 5:**
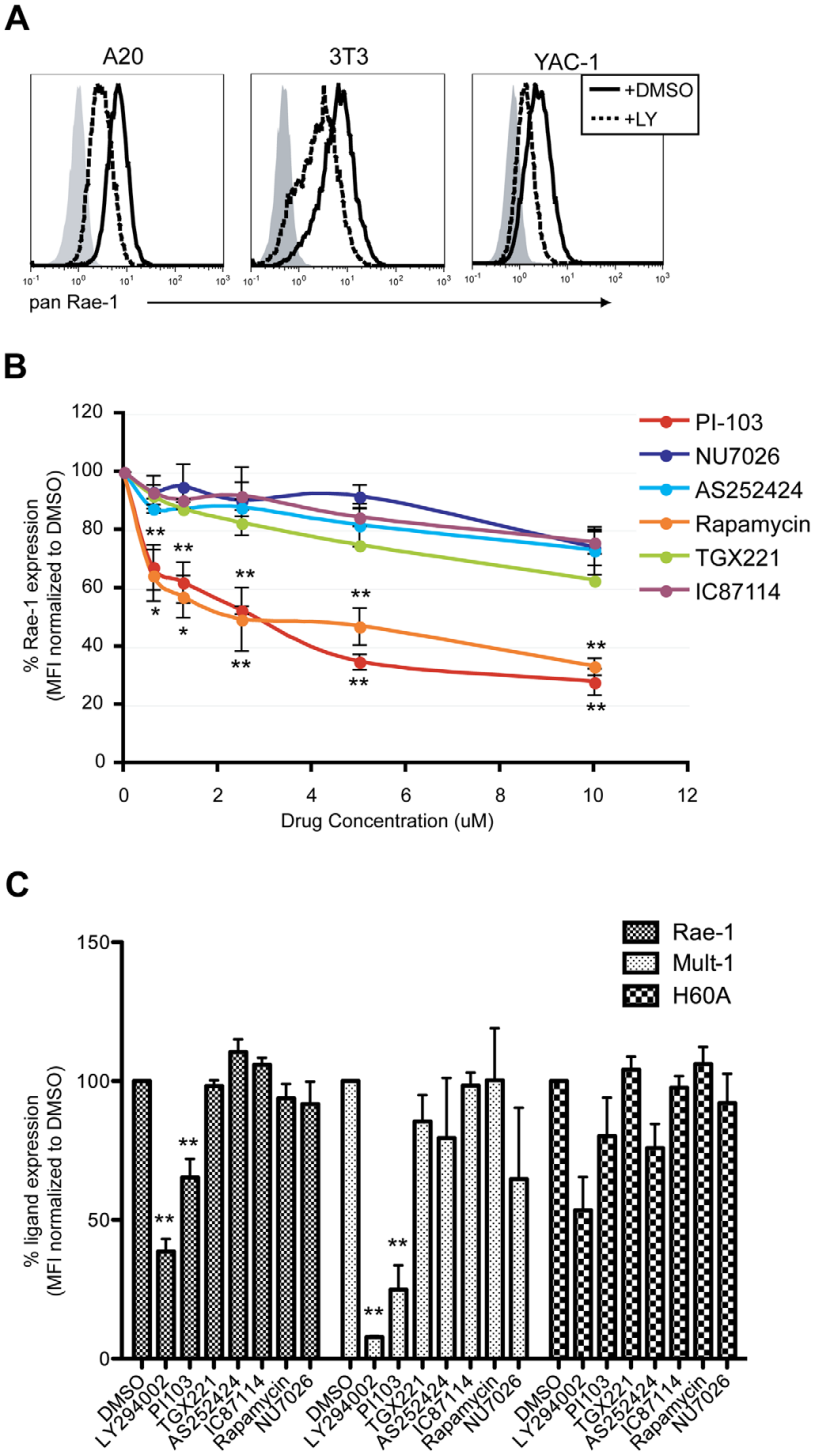
p110α PI3K is involved in the maintenance of RAE-1 and MULT-1 expression on transformed cells. **A**) A20, NIH 3T3 and YAC-1 tumor cells were treated with 20 uM LY294002 for 24 hrs and stained for RAE-1 at the cell surface. Histograms show isotype control (shaded gray), untreated (solid black) and treated cells (dashed black). The histogram is a representative figure from multiple independent experiments. **B**) YAC-1 cells were treated with the indicated inhibitors at 10 uM, 5 uM, 2.5 uM, 1.25 uM, and 0.625 uM and surface stained for RAE-1. SD and statistical significance were determined from three independent experiments. Statistical significance (** or *) on the bottom corresponds to PI-103 and the ones on top correspond to rapamycin treatments. **C**) NIH 3T3 cells expressing RAE-1, MULT-1, and H60a were treated with the indicated inhibitors for 24 hrs and stained for RAE-1, MULT-1 or H60a (20 uM LY294002, 1 uM PI-103, 1 uM TGX-221, 100 nM Rapamycin, 10 uM AS252424, 10 uM IC87114, and 10 uM NU7026). Percent RAE-1 and ligand expression were determined by normalizing the MFI of RAE-1 or other ligands in inhibitor-treated cells to the MFI of RAE-1 or other ligands in DMSO-treated cells. SD and statistical analyses were calculated from three independent experiments. **p<0.01 and *p<0.05.

The gene encoding p110α is an oncogene that is commonly mutated in human cancers [43], [44]. These mutations in p110α cause the PI3K pathway to be constitutively active, resulting in cellular transformation and oncogenesis [45]–[47]. Because RAE-1 molecules, along with other mouse and human NKG2D ligands, are frequently expressed on the surface of transformed cell lines as well as in some tumors *in vivo* [7], [8], we hypothesized that RAE-1 expression in transformed cells is also dictated by p110α PI3K signaling. To test this hypothesis, we first tested the effect of LY294002 treatment on three different types of transformed cell lines that all constitutively express RAE-1 at the cell surface: A20 (a B lymphoma cell line), NIH 3T3 (adherent fibroblast cell line) and YAC-1 (a T lymphoma cell line). Cell surface RAE-1 expression was susceptible to inhibition by LY294002 treatment in all three cell lines tested (Fig. 5A), suggesting that RAE-1 expression in these cells also depends on active PI3K.

To test the role of specific PI3K isoforms on RAE-1 expression in transformed cells, YAC-1 cells were treated with the same isoform-specific inhibitors used in Figure 4B and Figure S3, at a wide range of concentrations. RAE-1 surface expression was measured at 24 hours post-treatment. Similar to the effect observed in MCMV-infected cells, PI-103 treatment led to a significant decrease in the expression of RAE-1, whereas treatment with selective inhibitors of the other isoforms of PI3K (p110β, γ, and δ) or an inhibitor of DNA-PK had no significant effect on RAE-1 expression, even at high concentrations (Fig. 5B). In NIH 3T3 cells (Fig. 5C) and A20 cells (data not shown), we also observed inhibition of RAE-1 expression by PI-103, but not by inhibitors of other PI3K isoforms. The lack of response to inhibitors that target the p110β, γ, and δ isoforms of PI3K strongly indicates that signaling through p110α is specifically involved in the expression of RAE-1 in transformed cells as well. We also tested whether RAE-1 expression was impacted by rapamycin, an inhibitor of mTORC1, a downstream effector of PI3K. Whereas rapamycin did not inhibit RAE-1 expression in infected cells (Fig. 4D), NIH 3T3 cells (Fig. 5C) or A20 cells (data not shown), it did block RAE-1 expression in YAC-1 cells (Fig. 5B). These data suggest that mTORC1 plays a role in supporting RAE-1 expression in some transformed cells but not in others or in MCMV-infected fibroblasts.

In order to address whether the PI3K pathway regulates other mouse NKG2D ligands, we determined the effect of PI3K inhibition on expression of MULT-1 and H60a in NIH 3T3 cells, which constitutively express all three types of mouse NKG2D ligands. Similar to the results with RAE-1, MULT-1 expression was suppressed in NIH 3T3 cells treated with LY294002 or PI-103, but not with the other inhibitors (Fig. 5C). In contrast, H60a expression was not significantly affected by the inhibition of PI3K. Altogether the data illustrate a specific role of p110α PI3K in regulating RAE-1 and MULT-1 mouse NKG2D ligands in transformed cells.

## Discussion

Activation of NK cells through the interaction between NKG2D and NKG2D ligand is critical in the clearance of tumor cells, virally-infected cells, and stressed cells [1]. Although reports have implicated various effector molecules in regulating NKG2D ligand expression, the mechanism of ligand induction is still poorly understood. Here, we show that dysregulation of the PI3K pathway is a key signal required for expression of the RAE-1 family of NKG2D ligands during both infection and transformation and that it is further involved in the expression of another mouse NKG2D ligand, MULT-1.

In this study, we used both mouse fibroblasts and primary peritoneal macrophages to study the expression of the RAE-1 family members (Fig. 1 and Fig. S4). Because both macrophages and fibroblasts are infected by MCMV *in vivo*, the use of both of these cell types *in vitro* is informative [48]. We chose fibroblasts to perform all of the experiments because MCMVΔ152-infected macrophages do not express RAE-1 protein at the cell surface (Fig. S4C). The absence of surface expression occurred despite efficient infection of these cells, activation of the PI3K pathway, and a strong induction of RAE-1 mRNA, suggesting a cell type-specific block in a post-transcriptional step of RAE-1 biogenesis that does not prevent PI3K activation (Fig. S4A, B, and D).

### RAE-1 expression requires sensing of “patterns of pathogenesis”

Using fibroblasts, we observed that RAE-1 induction occurs only in infected cells (Fig. 1D) and that this induction requires active viral gene expression (Fig. 2). Because viral infections are often accompanied by production of defective viral particles that do not contain the entire viral genome, such specificity will presumably spare cells infected with defective viral particles from NK-cell mediated killing. This discrimination may be beneficial for the host because cells exposed to defective viral particles contain nucleic acids that function as pathogen associated molecular patterns (PAMPs) and activate innate immune sensors that induce production of type-I IFN and other proinflammatory molecules. Indeed, UV-inactivated MCMV is a potent activator of ISG15 (Fig. 2C). NK-mediated killing of these cells could potentially curtail inflammatory cytokine production. Additionally, cells infected with defective particles may also serve as good sources of antigens for cross-presentation by antigen presenting cells.

Recently, Vance et al. described the principle that signals associated with active bacterial infection and manipulation of the host cell machinery, termed “patterns of pathogenesis,” can serve to activate the innate immune response [49]. Here, we illustrated that UV-inactivated viral particles were sufficient to stimulate the IFN response, but expression of RAE-1 proteins was absolutely dependent on active infection and manipulation of the host cell machinery; activation of PI3K signaling being one example of such manipulation. Hence, PI3K activation appears to function as a “pattern of pathogenesis” for induction of RAE-1 expression. However, additional considerations described below, indicate that PI3K activation is not sufficient for Rae1 induction, suggesting that several signals may function cooperatively to induce RAE-1 expression.

### Multiple signals are required for the expression of RAE-1

We observed that RAE-1 expression requires viral genes that are expressed prior to viral replication, as demonstrated by the use of UV-inactivated virus and PAA (Fig. 2). This may suggest that viral early proteins are mediating the induction of RAE-1. Because immediate early proteins are the first to be expressed upon MCMV infection, we tested their role in the induction of RAE-1. Overexpression of GFP-fused MCMV ie1, ie2, and ie3 proteins alone or in combination did not lead to the induction of RAE-1 (Fig. S5), suggesting that although these proteins may play a role, they are not sufficient for RAE-1 expression.

In this manuscript, we focused on the involvement of cellular pathways that are activated during viral infection in the expression of RAE-1. The PI3K pathway regulates cellular functions including metabolism, cell cycle progression, proliferation, and apoptosis, and it is often dysregulated in infected and transformed cells [34], [50]. Class IA PI3K is generally activated through receptor tyrosine kinases (RTKs), which are receptors for growth factors, cytokines, and hormones. If class IA PI3K activation alone is sufficient to induce RAE-1 induction, it should occur in response to many cellular stimuli independently of infection. To test this, we stimulated cells for 24 hours with PDGF, which activates the well-studied RTK, PDGF receptor. Despite robust activation of the PI3K pathway, as illustrated by phosphorylation of Akt, RAE-1 induction did not occur (data not shown and Fig. S6A). Interestingly, PDGF-R has been shown to be a cellular receptor for HCMV [51]. Thus, the lack of RAE-1 induction with PDGF treatment is consistent with our finding that viral gene expression is absolutely necessary. Additionally, overexpression of a constitutively active form of p110α, p110α H1047R, by itself was insufficient to induce RAE-1 expression, again despite robust activation of the PI3K pathway (Fig. S6B and 3C).

Together these results strongly suggest that RAE-1 induction is tightly regulated such that expression of the main viral immediate early proteins (ie1, ie2, and ie3) or activation of class IA PI3K in the absence of infection are not sufficient to induce ligand induction and that additional signals are likely required for the induction. Future investigations are necessary to identify these additional pathways that are necessary for RAE-1 induction.

### The role of PI3K activation in regulating RAE-1 expression

To determine whether PI3K activation is involved in regulating RAE-1 at the post-translational level, we stably expressed the coding region of RAE-1α or RAE-1γ isoform from an exogenous promoter. The choice of these isoforms came from our observation that they were induced upon MCMV infection at the RNA level (Fig. S8A) and at the protein level, confirmed by the use of two different mutant viruses lacking m152 (Fig. S8B and C). RAE-1 expression in these cells was not affected by LY294002 treatment, arguing that trafficking of RAE-1 and sustained expression of mature RAE-1 proteins at the cell surface is not dependent on PI3K activation (Fig. S7B).

Notably, although LY294002 treatment of infected cells resulted in a complete loss of RAE-1 expression at the cell surface (Fig. 3A), RAE-1 mRNA was still induced five fold compared to uninfected cells (Fig. S7A). Therefore, it is possible that the PI3K pathway plays a role in regulating RAE-1 at least in part at the post-transcriptional level, prior to trafficking to the surface. Activation of PI3K can enhance cellular translation via formation of the translation initiation complex containing eIF4E [52], raising the possibility that RAE-1 is regulated at the translational level upon PI3K activation. Nevertheless, LY294002 treatment reduced the amount of RAE-1 mRNA in infected cells by three fold, suggesting a possible role of PI3K in either transcription of the RAE-1 gene or stabilization of RAE-1 mRNA as well. It has recently been shown that transcripts of two human NKG2D ligands, MICA and MICB, are regulated by several cellular microRNAs [20], raising an additional possibility that PI3K regulates microRNAs that target RAE-1.

### The importance of PI3K signaling in transformed cells and cancer cells

The gene encoding the p110α catalytic subunit of PI3K is often mutated in mouse and human tumors so as to render PI3K constitutively active, and thus functions as an oncogene [53]. Despite the involvement of p110β, γ, and δ isoforms in cancer, p110α is the only catalytic subunit of PI3K found to be mutated in tumors, suggesting a unique role of p110α in cellular transformation [41]. We observed that p110α PI3K is important for the expression of RAE-1 in multiple transformed cell lines (Fig. 5). The mechanism by which the different PI3K heterodimers mediate their non-redundant functions is poorly understood [54]. It is possible that the specificity of p110α is achieved at the cell surface receptor level (i.e. RTKs or GPCRs) or by one of the downstream effectors of PI3K. Nonetheless, the role of p110α PI3K in regulating RAE-1 expression during both viral infection and transformation is an intriguing finding that deserves further investigation.

In this study, we also observed an effect of PI3K inhibition on MULT-1 expression, but not H60a. The observed difference in the requirement for PI3K signaling for expression of different mouse NKG2D ligands is interesting and may have several explanations. One possibility is that the difference reflects a specialization in NKG2D ligands, in which RAE-1 and MULT-1 respond to activated PI3K, whereas H60a responds to distinct cellular cues or stress pathway mediators. The notion that NKG2D ligands respond to different stress pathways was already suggested by the finding that MULT-1 is unique among the mouse NKG2D ligands in being regulated by stress associated with heat shock or UV irradiation [22]. The possibility that H60a has unique regulatory properties is also suggested by the sequence of its 3′ untranslated region (UTR), which is unusually long (3kb) in comparison to those of RAE-1 and MULT-1 (400bp and 700bp, respectively), suggesting that it may contain a unique set of regulatory elements.

PI3K and its downstream mediators such as Akt and mTORC1 have been key targets in the development of cancer therapies [50]. In particular, chemical analogues of the inhibitors used in this study are used in clinical studies as therapeutics for cancer. As cancer drug development progresses, it will be important to take into consideration the potential for these PI3K inhibitors to greatly diminish NK-cell recognition and cytolysis of targets; especially because NK cells are important for recognition and clearance of tumor cells [2].

Here, we identified a common pathway between infected and transformed cells that is required for expression of the RAE-1 family of mouse NKG2D ligands. The results of this study are the first to demonstrate the role of the PI3K pathway in the expression of NKG2D ligands or other events that sensitize cells for immune recognition. Our data suggest that PI3K dysregulation in the context of disease is a key signal sensed by cells for expression of RAE-1. This study provides an important direction for future investigations designed to elucidate how NKG2D ligand expression is regulated and how it is restricted to diseased cells.

## Materials and Methods

### Cells

Established tail-derived fibroblasts were prepared as described previously [18]. Established fibroblasts, BALB/c 3T3 (ATCC, CCL-163), NIH 3T3 (ATCC, CRL-1658), and BOSC (ATCC, CRL-11270) cells were maintained in DMEM with 5% FBS and 1% penicillin and streptomycin. YAC-1s (ATCC, TIB-160) and A20s were maintained in RPMI. Peritoneal macrophages obtained from C57BL/6 mice were cultured overnight in RPMI with 10% MCSF provided by Dr. Portnoy (UC Berkeley), 10% FBS, and 1% penicillin and streptomycin.

### Virus propagation, infection and titering

MCMVΔ152 and Δ152-rev viruses were generously provided by Dr. Hill (Oregon Health and Science University, Oregon). MCMVΔ152-GFP virus was kindly provided by Dr. Jonjic (University of Rijeka, Croatia). MCMVWT (Smith strain) and MCMVΔm04+m06+m152 viruses were generously provided by Dr. Koszinowski (Max von Pettenkofer-Institute, Munich, Germany). All viruses were propagated in NIH 3T3 cells and titered in BALB/c 3T3 cells. For all infection experiments, fibroblasts were infected at MOI of 5, input virus removed at 2 hrs pi, and infection was allowed to take place for a total of 24 hrs. Supernatants were collected at the time of harvest at 24 hrs pi and used for titering in BALB/c 3T3s. For UV-inactivation of the virus, viral supernatant was placed directly under the UV light in a sterile tissue culture hood for 30 minutes. To confirm successful UV-inactivation, MCMV e1 gene was PCR amplified from viral genomic DNA isolated from equal volumes of untreated or UV-treated virus stock. Briefly, viral DNA was extracted from viral supernatants by adding an equal volume of phenol/chloroform followed by another round of chloroform extraction, and isopropanol was used to precipitate the DNA. UV-inactivation was further confirmed by performing a plaque assay on supernatants obtained from cells infected with either untreated or UV-treated virus.

### Flow cytometry

Fibroblasts and 3T3 cells were harvested in 2 mM EDTA PBS and stained with monoclonal anti-mouse pan-specific RAE-1, RAE-1α/β/γ, RAE-1β/δ, RAE-1ε, MULT-1, H60A or Rat IgG_2A_ isotype control (all purchased from R&D) followed by PE-conjugated goat anti-rat IgG (Jackson ImmunoResearch Laboratories). YAC-1s, A20s, and peritoneal macrophages were first incubated with an anti-mouse CD16/CD32 FcBlock (BD), followed by pan RAE-1 antibody and FITC or PE-conjugated anti-rat IgG_2A_ antibody (BD). All samples were co-stained with 7-AAD (BD). MCMV m157-specific monoclonal antibody (6H1.2.1) was generously provided by Dr. Yokoyama (Washington University School of Medicine, MO).

### Reverse transcription quantitative real-time PCR

RNA from fibroblasts and macrophages were extracted in Trizol (Invitrogen), treated with RQ1 DNase (Promega), and total RNA was reverse transcribed using oligo(dT)_15_ primer (Integrated DNA Technologies) and SuperScriptII (Invitrogen) at 42°C for 50 minutes. cDNAs were analyzed using ABI7300 Real Time PCR System. RAE-1 isoform specific primers were described previously [55]. Primers for RAE-1 and ISG15 are described in the Table S1. cDNAs from uninfected tail fibroblasts were used to amplify regions within the catalytic and regulatory domains of class I PI3K using primers described in the Table S1.

### Inhibitor treatments

Inhibitors for all infection experiments were added to the media 2 hrs pi to first allow viral attachment and entry, and they were left in the culture media for the remainder of the 24 hr infection. Phosphonoacetic acid (PAA) was purchased from Sigma Aldrich and used at a final concentration of 100 ug/ml, pH 7.4. YAC-1s were cultured in the presence of PI3K inhibitors for 18 hrs. U0126, SB203580, SB600125, SB218078, UCN-01, LY294002, Rapamycin, and NU7026 were purchased from Calbiochem. PI3Kαi2, PI-103, TGX-221, and AS252424 were purchased from Cayman Chemicals. Caffeine was purchased from Sigma, and IC87114 was kindly provided by Dr. Okkenhaug (Babraham Institute, Cambridge, UK). The final concentrations of all of these inhibitors are stated in the figure legends of the corresponding figures.

### Constructs and transduction

The coding region of RAE-1α or γ isoforms was cloned into a retroviral vector, pBMN-IN. p110α H1047R was cloned into a retroviral vector pMG-hygro. Retroviral supernatants were obtained as described previously [56]. MCMV ie1, 2, and 3 fused to GFP was cloned into pEGFP.N1 (Clontech) and transiently transfected using Lipofectamine 2000 (Invitrogen).

### Western blotting

Mouse fibroblasts or peritoneal macrophages were serum-starved overnight and infected with MCMV in the presence of DMSO or LY294002 or treated with PDGF (Sigma) for 24 hrs. Fibroblasts transduced with empty vector or transduced with p110α H1047R were serum-starved overnight. Cell lysates were analyzed by western blotting for phospho-Akt S473 and Akt according to manufacturer's instructions (Cell Signaling). The relative ratio of P-Akt to total Akt was determined using ImageJ.

### Statistical analysis

A two-tailed, paired student t-test was performed on all samples where statistical significance is indicated.

### Ethics statement

All animals were handled in strict accordance with good animal practice as defined by the Panel on Euthanasia of the American Veterinary Society. We have received approval for these experiments from the UC Berkeley IACUC (R292).

## Supporting Information

Figure S1 **MCMV**Δ**m04+m06+m152-mediated induction of RAE-1 is independent of the DNA damage pathway.** RAE-1 surface expression was determined in fibroblasts infected with MCMV lacking m04, m06, and m152 (MCMVΔm04+m06+m152) in the presence of 150 nM SB218078, 70 nM UCN-01 or 5 mM Caffeine. The data shown are gated on live, 7AAD negative cells. Histograms show isotype control (shaded gray), uninfected (solid gray), MCMVΔm04+m06+m152-infected (solid black), and MCMVΔm04+m06+m152-infected cells in the presence of inhibitors (dashed black).
(PDF)

Figure S2 **Erk and p38/Jnk activation is not required for MCMV-mediated induction of RAE-1.** Fibroblasts infected with MCMVΔm04+m06+m152 in the presence of 2 uM U0126 or 1 uM SB203580 and SB600125 were stained with the pan RAE-1 antibody. The data shown are gated on live, 7AAD negative cells. Histograms show isotype control (shaded gray), uninfected (solid gray), MCMVΔ3-infected (solid black), and MCMVΔm04+m06+m152-infected cells in the presence of inhibitors (dashed black).
(PDF)

Figure S3 **p110**α **PI3K is specifically involved in the induction of RAE-1 in fibroblasts infected with MCMV.** Fibroblasts infected with MCMVΔm04+m06+m152 in the presence of the indicated inhibitors at 10 uM, 5 uM, 2.5 uM, 1.25 uM, and 0.625 uM were stained for RAE-1. The percent RAE-1 expression was determined by normalizing the MFI of RAE-1 in cells that were treated with the inhibitors to the MFI of RAE-1 in cells that were treated with DMSO. SD and statistical significance were determined from three independent experiments. **p<0.01.
(PDF)

Figure S4 **MCMV infection of peritoneal macrophages does not induce RAE-1 surface expression.** **A**) Peritoneal macrophages from C57BL/6 mice were infected with GFP expressing MCMVWT (WT-GFP) or MCMVΔ152 (Δ152GFP) at moi 1 for 24 hrs. The level of GFP expression was determined by flow cytometry. **B**) RAE-1 mRNA level in peritoneal macrophages infected with MCMVΔ152 (Δ152), MCMVΔ152-revertant (Δ152-rev), MCMVΔ152-GFP (Δ152-GFP) or MCMVWT (WT) was quantified by RT-qPCR and was normalized to the level of HPRT for each sample. The data represent fold induction over uninfected cells (NI). SD and statistical significance were determined based on three independent experiments. **p<0.01. **C**) RAE-1 surface expression in peritoneal macrophages infected with Δ152 or Δ152-rev was determined by flow cytometry using a pan RAE-1 antibody. **D**) Cellular lysates were obtained from peritoneal macrophages infected with Δ152 or Δ152-rev for 24 hrs in the presence of DMSO or LY294002 and used to probe for Akt phosphorylation at serine 473 or total Akt. The blot is a representative figure of three independent experiments.
(PDF)

Figure S5 **Expression MCMV immediate early genes is not sufficient to induce RAE-1 induction.** Fibroblasts were transiently transfected with GFP-fused MCMV ie1, 2, and 3 alone or in combination (ie1+2+3) for 24 hrs and stained for RAE-1. The data shown were gated on live, 7AAD negative cells.
(PDF)

Figure S6 **Activation of PI3K by PDGF or overexpression of a constitutively active form of p110α does not induce RAE-1 expression.** Fibroblasts treated with (**A**) 20 ng/ml of PDGF for 24 hrs or (**B**) stably expressing p110α H1047R (constitutively active) were stained with the pan RAE-1 antibody. The data shown are gated on live, 7AAD negative cells. Histograms show isotype control (shaded gray), untreated (solid black), and treated or empty vector (dashed black).
(PDF)

Figure S7 **PI3K pathway is involved in post-transcriptional regulation of RAE-1 upon MCMV infection.** **A**) The level of RAE-1 mRNA was determined in fibroblasts infected with wildtype MCMV in the presence (WT+LY) or absence (WT) of LY294002 using RT-qPCR at 24 hrs pi. Fold induction was determined by normalizing the infected samples to uninfected samples (NI). SD was determined from three independent experiments. **B**) Fibroblasts stably transduced with RAE-1α or γ were treated with LY294002 for 24 hrs and stained with the RAE-1αβγ-specific antibody. Histograms show isotype control (shaded gray), DMSO-treated (solid black), and LY294002-treated cells (dashed black).
(PDF)

Figure S8 **Expression of RAE-1 isoforms is differentially controlled in mouse fibroblasts infected with MCMV.** (**A**) RNA extracted from uninfected fibroblasts (NI) or fibroblasts infected with MCMVΔm04+m06+m152 (MCMVΔ3) were used to quantify the expression levels of the RAE-1 isoforms using RT-qPCR. Fold induction was determined by normalizing the infected samples to uninfected samples (NI). SD was determined from two independent experiments. ND; undetectable. Fibroblasts infected with MCMVΔ3 (**B**) or MCMVΔ152 (**C**) were surfaced stained with RAE-1 α/β/γ, β/δ or ε antibody at 24 hrs pi. The data shown were gated on live, 7AAD negative cells. Histograms show isotype control (shaded gray), uninfected (dashed black), and infected cells (solid black). The values represent the percent of live RAE-1 positive cells.
(PDF)

Table S1 **Primer sequences and product sizes.** The table contains primer sequences for all of the indicated target genes and the corresponding expected product size for each primer set.
(DOC)
