# Supplementary figures and images for: Expression of the RAE-1 Family of Stimulatory NK-Cell Ligands Requires Activation of the PI3K Pathway during Viral Infection and Transformation
Source: PLoS Pathog. 2011 Sep 22;7(9):e1002265. doi: 10.1371/journal.ppat.1002265 (PMC3178570; doi:10.1371/journal.ppat.1002265)

Supplementary Figure 1

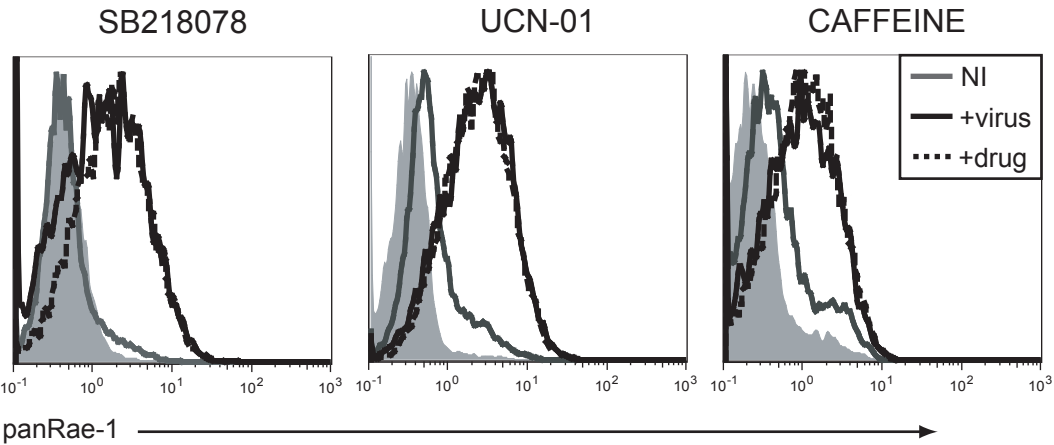

Supplement: Figure S1 — MCMVΔm04+m06+m152-mediated induction of RAE-1 is independent of the DNA damage pathway. RAE-1 surface expression was determined in fibroblasts infected with MCMV lacking m04, m06, and m152 (MCMVΔm04+m06+m152) in the presence of 150 nM SB218078, 70 nM UCN-01 or 5 mM Caffeine. The data shown are gated on live, 7AAD negative cells. Histograms show isotype control (shaded gray), uninfected (solid gray), MCMVΔm04+m06+m152-infected (solid black), and MCMVΔm04+m06+m152-infected cells in the presence of inhibitors (dashed black). (PDF) [file ppat.1002265.s001.pdf]

Supplementary Figure 2

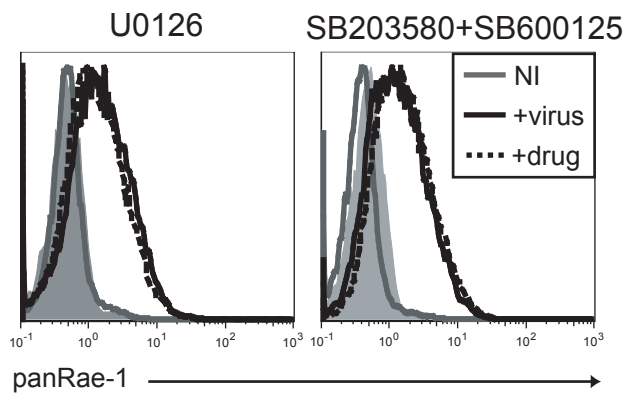

Supplement: Figure S2 — Erk and p38/Jnk activation is not required for MCMV-mediated induction of RAE-1. Fibroblasts infected with MCMVΔm04+m06+m152 in the presence of 2 uM U0126 or 1 uM SB203580 and SB600125 were stained with the pan RAE-1 antibody. The data shown are gated on live, 7AAD negative cells. Histograms show isotype control (shaded gray), uninfected (solid gray), MCMVΔ3-infected (solid black), and MCMVΔm04+m06+m152-infected cells in the presence of inhibitors (dashed black). (PDF) [file ppat.1002265.s002.pdf]

Supplementary Figure 3

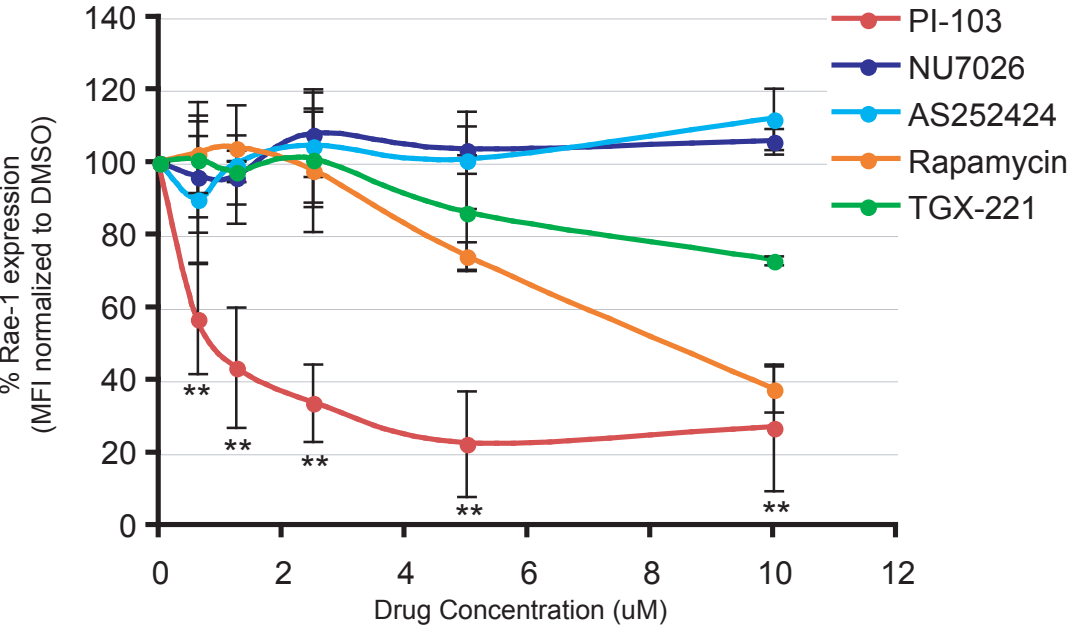

Supplement: Figure S3 — p110α PI3K is specifically involved in the induction of RAE-1 in fibroblasts infected with MCMV. Fibroblasts infected with MCMVΔm04+m06+m152 in the presence of the indicated inhibitors at 10 uM, 5 uM, 2.5 uM, 1.25 uM, and 0.625 uM were stained for RAE-1. The percent RAE-1 expression was determined by normalizing the MFI of RAE-1 in cells that were treated with the inhibitors to the MFI of RAE-1 in cells that were treated with DMSO. SD and statistical significance were determined from three independent experiments. **p<0.01. (PDF) [file ppat.1002265.s003.pdf]

Supplementary Figure 4

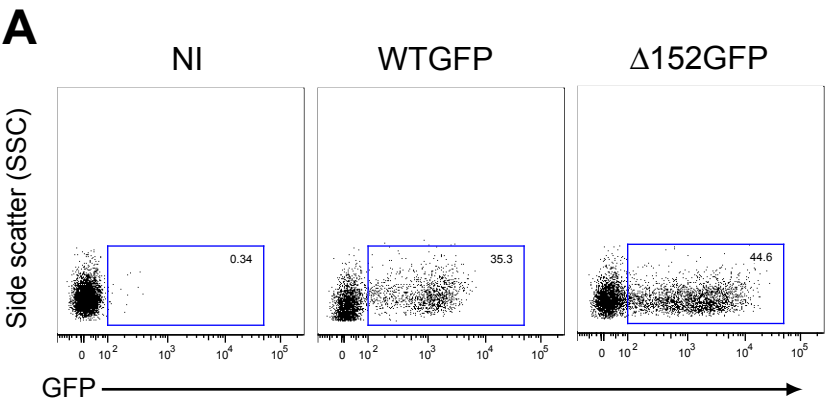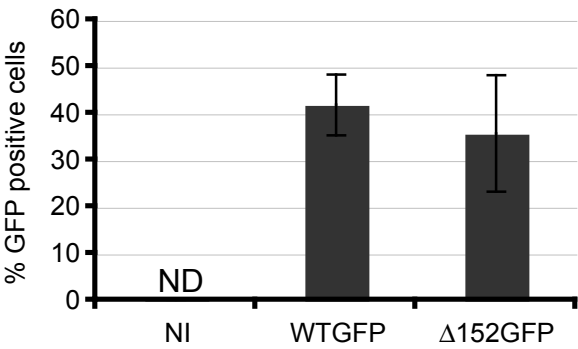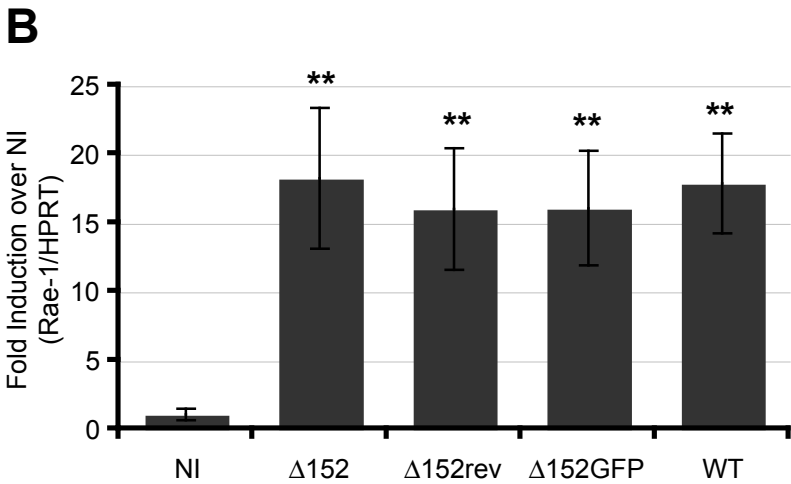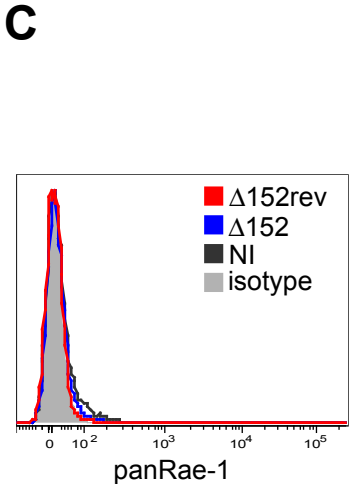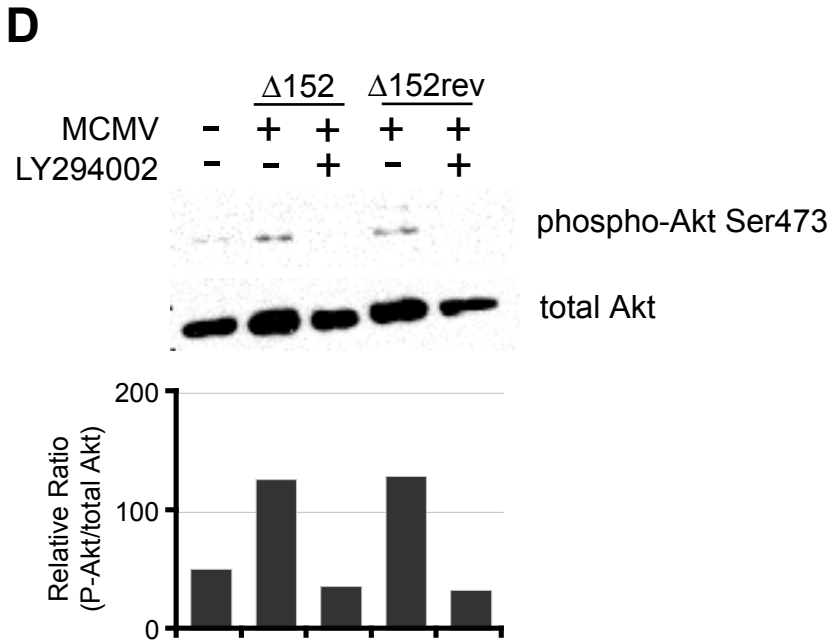

Supplement: Figure S4 — MCMV infection of peritoneal macrophages does not induce RAE-1 surface expression. A) Peritoneal macrophages from C57BL/6 mice were infected with GFP expressing MCMVWT (WT-GFP) or MCMVΔ152 (Δ152GFP) at moi 1 for 24 hrs. The level of GFP expression was determined by flow cytometry. B) RAE-1 mRNA level in peritoneal macrophages infected with MCMVΔ152 (Δ152), MCMVΔ152-revertant (Δ152-rev), MCMVΔ152-GFP (Δ152-GFP) or MCMVWT (WT) was quantified by RT-qPCR and was normalized to the level of HPRT for each sample. The data represent fold induction over uninfected cells (NI). SD and statistical significance were determined based on three independent experiments. **p<0.01. C) RAE-1 surface expression in peritoneal macrophages infected with Δ152 or Δ152-rev was determined by flow cytometry using a pan RAE-1 antibody. D) Cellular lysates were obtained from peritoneal macrophages infected with Δ152 or Δ152-rev for 24 hrs in the presence of DMSO or LY294002 and used to probe for Akt phosphorylation at serine 473 or total Akt. The blot is a representative figure of three independent experiments. (PDF) [file ppat.1002265.s004.pdf]

Supplementary Figure 5

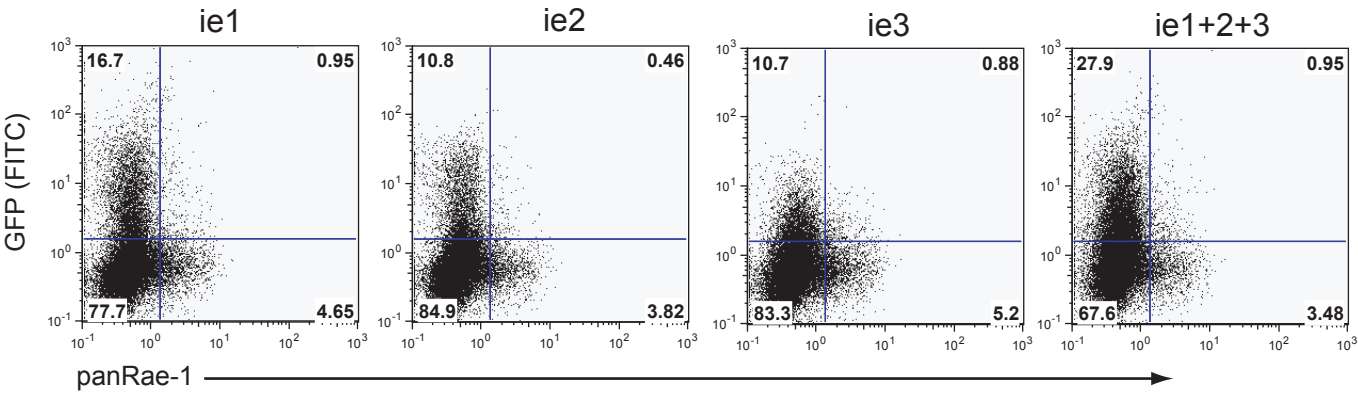

Supplement: Figure S5 — Expression MCMV immediate early genes is not sufficient to induce RAE-1 induction. Fibroblasts were transiently transfected with GFP-fused MCMV ie1, 2, and 3 alone or in combination (ie1+2+3) for 24 hrs and stained for RAE-1. The data shown were gated on live, 7AAD negative cells. (PDF) [file ppat.1002265.s005.pdf]

Supplementary Figure 6

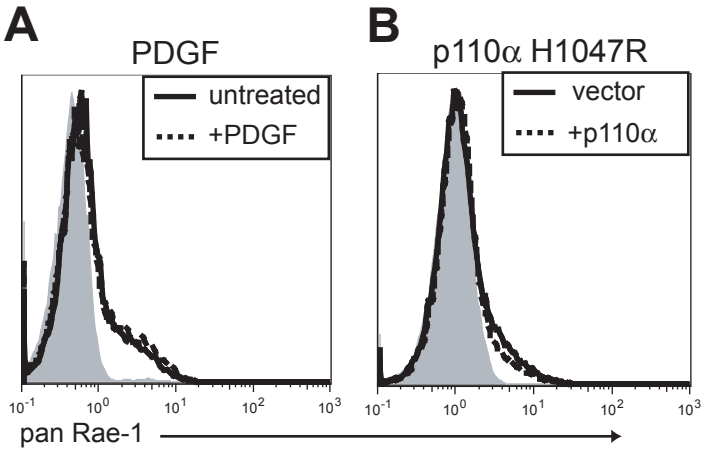

Supplement: Figure S6 — Activation of PI3K by PDGF or overexpression of a constitutively active form of p110α does not induce RAE-1 expression. Fibroblasts treated with (A) 20 ng/ml of PDGF for 24 hrs or (B) stably expressing p110α H1047R (constitutively active) were stained with the pan RAE-1 antibody. The data shown are gated on live, 7AAD negative cells. Histograms show isotype control (shaded gray), untreated (solid black), and treated or empty vector (dashed black). (PDF) [file ppat.1002265.s006.pdf]

Supplementary Figure 7

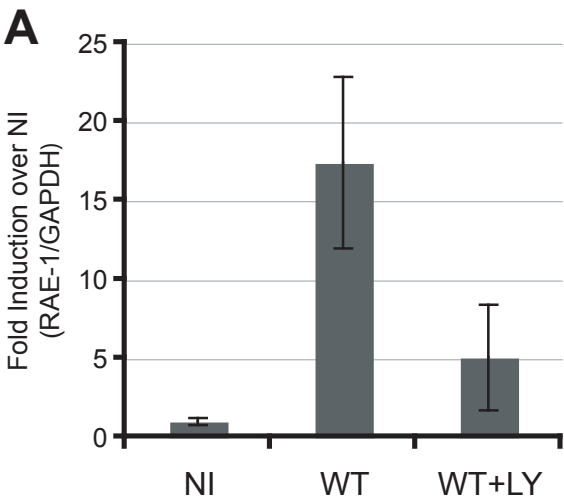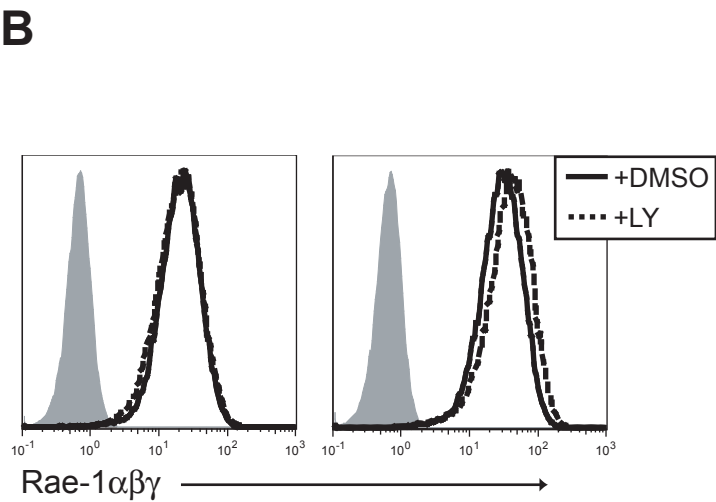

Supplement: Figure S7 — PI3K pathway is involved in post-transcriptional regulation of RAE-1 upon MCMV infection. A) The level of RAE-1 mRNA was determined in fibroblasts infected with wildtype MCMV in the presence (WT+LY) or absence (WT) of LY294002 using RT-qPCR at 24 hrs pi. Fold induction was determined by normalizing the infected samples to uninfected samples (NI). SD was determined from three independent experiments. B) Fibroblasts stably transduced with RAE-1α or γ were treated with LY294002 for 24 hrs and stained with the RAE-1αβγ-specific antibody. Histograms show isotype control (shaded gray), DMSO-treated (solid black), and LY294002-treated cells (dashed black). (PDF) [file ppat.1002265.s007.pdf]

Supplementary Figure 8

**A**

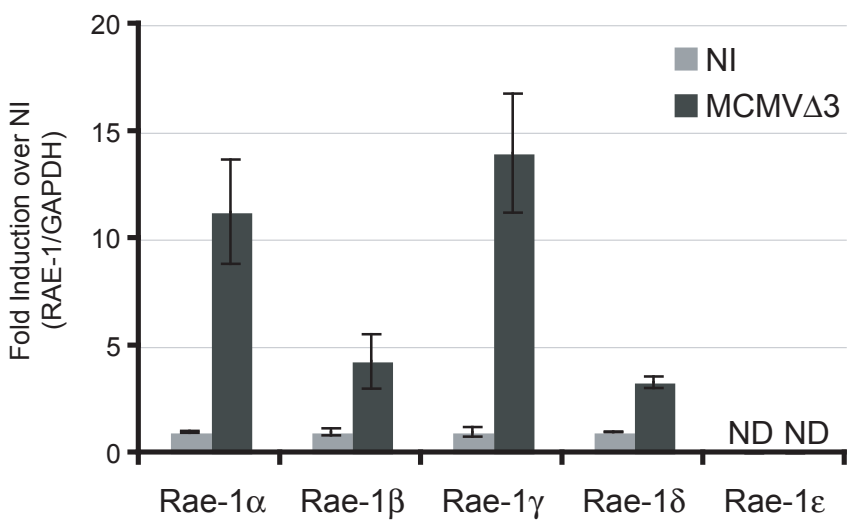

**B**

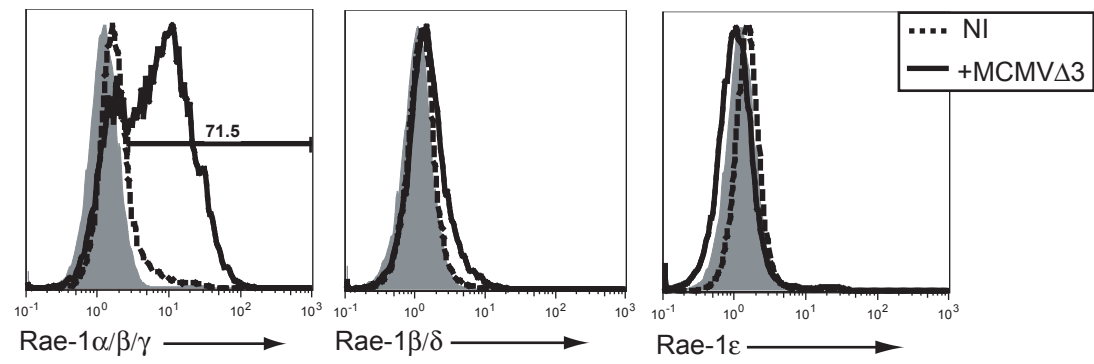

**C**

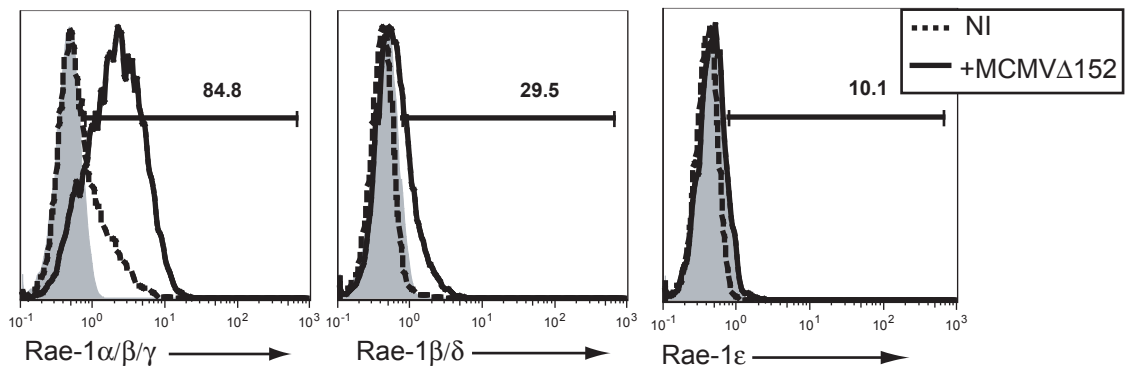

Supplement: Figure S8 — Expression of RAE-1 isoforms is differentially controlled in mouse fibroblasts infected with MCMV. (A) RNA extracted from uninfected fibroblasts (NI) or fibroblasts infected with MCMVΔm04+m06+m152 (MCMVΔ3) were used to quantify the expression levels of the RAE-1 isoforms using RT-qPCR. Fold induction was determined by normalizing the infected samples to uninfected samples (NI). SD was determined from two independent experiments. ND; undetectable. Fibroblasts infected with MCMVΔ3 (B) or MCMVΔ152 (C) were surfaced stained with RAE-1 α/β/γ, β/δ or ε antibody at 24 hrs pi. The data shown were gated on live, 7AAD negative cells. Histograms show isotype control (shaded gray), uninfected (dashed black), and infected cells (solid black). The values represent the percent of live RAE-1 positive cells. (PDF) [file ppat.1002265.s008.pdf]
