# Supplementary material for: Expression of the RAE-1 Family of Stimulatory NK-Cell Ligands Requires Activation of the PI3K Pathway during Viral Infection and Transformation
Source: PLoS Pathog. 2011 Sep 22;7(9):e1002265. doi: 10.1371/journal.ppat.1002265 (PMC3178570; doi:10.1371/journal.ppat.1002265)
Supplement: Table S1 — Primer sequences and product sizes. The table contains primer sequences for all of the indicated target genes and the corresponding expected product size for each primer set. (DOC) [file ppat.1002265.s009.doc]

**Table S1**

| **Target gene** | **Sequence (5’-3’)** | **Product Size (bp)** |
| --- | --- | --- |
| Rae-1 | **F:**ACCCGAATGCAGACAGGAAGTTGA | 72 |
|  | **R:**GGACCTTGAGGTTGATCTTGGCTT |  |
| GAPDH | **F:**GAAGGTCGGTGTGAACGGA | 240 |
|  | **R:** GTTAGTGGGGTCTCGCTCCT |  |
| HPRT | **F:**CTGGTGAAAAGGACCTCTCG | 108 |
|  | **R:**TGAAGTACTCATTATAGTCAAGGGCA |  |
| ISG15 | **F:**TCCATGACGGTGTCAGAACT | 123 |
|  | **R:**GACCCAGACTGGAAAGGGTA |  |
| p110α | **F:**AAAGGCCACTGTGGTTGAATTGGG | 536 |
|  | **R:**CAAATGGCACACGTTCCCGCTTAT |  |
| p110β | **F:**TGCTTTCCAAACAGGTGGAAGCAC | 494 |
|  | **R:**TGGCAGCCACGTTACTACTGTTCA |  |
| p110γ | **F:**AGCCAGCGCATCAGCAAGA | 529 |
|  | **R:**CGATGAAGATGCAGTTGTTGGCAATCTTT |  |
| p110δ | **F:**ACTGTGTCAAGCTCGGAGGTGAAT | 618 |
|  | **R:**TCTGGGCCACATCCTCGTGTTTAT |  |
| p85α/p55α/ p50α | **F:**AAGAACAATGCCAAACCCAGGAGC | 630 |
|  | **R:**TGCACGAGGGAGGTGTGTTGATAA |  |
| p85β | **F:**ATCAAAGTCTTCCACCGGGATGGT | 570 |
|  | **R:**TGAGGTCAGGTTTGAGGCTGTTCA |  |
| p55γ | **F:**GACAAATTGCGGGACATGCCAGAT | 426 |
|  | **R:**AGGCTTCAATGGCAGTCCTCTTCA |  |
| p101 | **F:**TCCACACTACGGGTTGTGGTCTTT | 799 |
|  | **R:**ACCACTTCTGTCAGGGTTAGCGTT |  |
| NheI-Rae1-KpnI | **F:**ATCGGCTAGCATGGCCAAGG | * |
|  | **R:**CGATGGTACCTTTCCTCTTCACATTGCA |  |
| MCMV e1 | **F:** TCGAAGAGGAATGTTCTCCACGCA | 175 |
|  | **R:** GGACAGCGAACTAGCAGCATAACT |  |

F: forward primer sequence

R: reverse primer sequence

*The product size for this primer depends on the different Rae-1 isoforms. For each isoform, the final product would be an additional 20 nucleotides to account for the restriction sites and extra flanking sequences.
